# Sleep Disturbances in Autoimmune Neurologic Diseases: Manifestation and Pathophysiology

**DOI:** 10.3389/fnins.2021.687536

**Published:** 2021-08-06

**Authors:** Dou Yin, Sheng Chen, Jun Liu

**Affiliations:** Department of Neurology, Institute of Neurology, Ruijin Hospital Affiliated to Shanghai Jiao Tong University School of Medicine, Shanghai, China

**Keywords:** sleep disturbances, autoimmune encephalitis, antibody, insomnia, narcolepsy

## Abstract

Autoimmune neurologic diseases are a new category of immune-mediated disease demonstrating a widely varied spectrum of clinical manifestations. Recently, sleep disturbances in patients with autoimmune neurologic diseases have been reported to have an immense negative impact on the quality of life. Excessive daytime sleep, rapid eye movement sleep behavior disorder (RBD), and narcolepsy are the most frequent sleep disorders associated with autoimmune neurologic diseases. Sleep disturbances might be the initial symptoms of disease or persist throughout the course of the disease. In this review, we have discussed sleep disturbances in different autoimmune neurologic diseases and their potential pathophysiological mechanisms.

## Introduction

Sleep is one of the most important physiologic functions and sleep disturbances are common in neurodegenerative diseases such as Parkinson’s disease and Alzheimer’s disease. Studies from sleep centers have found that at least 40%–65% of patients with idiopathic rapid eye movement sleep behavior disorder (RBD) will develop a defined neurodegenerative phenotype over 10 years, indicating that RBD may be a biomarker of neurodegenerative diseases (Postuma et al., 2013). Autoimmune neurologic diseases are a new category of disease associated with antibodies against neuronal cell surfaces or synaptic proteins. The antigens are ion channels, cell-adhesion molecules and ligand-gated receptors. These antigens are distributed at different levels throughout the brain, with patients manifesting focal or general clinical features. Autoimmune neurologic diseases, such as autoimmune encephalitis, can cause several neurological symptoms, such as memory deterioration, seizures, psychosis and abnormal movement. Recently, sleep dysfunction has been frequently reported in these patients. Moreover, some studies have reviewed sleep disorders in autoimmune encephalitis (Blattner and Day, 2020; Iranzo, 2020; Munoz-Lopetegi et al., 2020) and autoimmune neurological syndromes (Devine and St Louis, 2021). Sleep disorders present as excessive daytime sleep, RBD, sleep-related breathing disorders, non-rapid eye movement (NREM) sleep parasomnias or narcolepsy. These manifestations might be the initial symptoms of disease or persist throughout the course of the disease. In this review, we discuss various sleep dysfunctions and their mechanisms in autoimmune neurological disorders.

## Anti-*N*-Methyl-*D*-Aspartate Receptor Encephalitis

Anti-*N*-methyl-D-aspartate receptor (Anti-NMDAR) encephalitis is one of the most common subtypes of autoimmune encephalitis and preponderantly affects young women and children with or without teratomas (Florance et al., 2009). This subtype is characterized by serum and cerebrospinal fluid (CSF) immunoglobulin G (IgG) antibodies against the NR1 subunit of the *N*-methyl-D-aspartate receptor (NMDAR), presenting with psychiatric and cognitive symptoms, movement disorders, seizures, sleep disorders, autonomic failure and changes in consciousness (Dalmau et al., 2008). An observational cohort study reported that 38% of patients had an underlying neoplasm, most of which were ovarian teratomas. Brain magnetic resonance imaging (MRI) and electroencephalography (EEG) results were found to be abnormal in 33 and 90% of patients, respectively (Titulaer et al., 2013), which are usually indicative of non-specific, slow and disorganized activity, sometimes accompanied by electrographic seizures (Dalmau et al., 2011). Extreme delta brush, characterized by rhythmic delta activity at 1–3 Hz with superimposed bursts of rhythmic 20–30 Hz beta frequency activity “riding” on each delta wave, was found in 30% of patients and was associated with more prolonged hospitalization and a trend toward worse outcomes (Schmitt et al., 2012). A study has reported sleep disturbances such as insomnia, hypersomnia, sleep-wake reversal, bad dreams/night terrors, and decreased need for sleep in 21% of patients (Al-Diwani et al., 2019). A systematic review reported that 39.5% of patients had insomnia, a condition that presents very early in the course of illness (Gurrera, 2019). Erratic sleep patterns, insomnia, and, less frequently, hypersomnia have been reported in children and adolescent patients (Florance et al., 2009). Another study has reported that approximately 27% of patients have prominent sleep dysfunction, including hypersomnia and inversion of sleep patterns after recovery (Dalmau et al., 2008). In some cases, sleep disturbance (insomnia) precedes the onset of dyskinesia and persists for a long time, even after dyskinesia subsides (Poloni et al., 2010), which suggests primary sleep disruption rather than effects of mental confusion, motor agitation or dyskinesia. A prospective observational single-centre study based on video-polysomnography (PSG) and neuropsychological evaluation revealed a temporal pattern of sleep disturbances in anti-NMDAR encephalitis (Arino et al., 2020). In the acute phase, 89% of patients reported insomnia, and after the acute stage, 78% were found to have hypersomnia. Sedatives, with a mean benefit of 67.4% per medication, were the most effective drugs for symptomatic treatment (Mohammad et al., 2016).

The disease pathophysiology is complex and remains undefined. NMDARs are ligand-gated excitatory ion channels, located in the cortical neurons, hippocampus and cerebellum. NMDARs play a key role in regulating central nervous system (CNS) synaptic excitation and plasticity. NR1 subunit of the NMDAR is the target of anti-NMDAR antibodies. A cell electrophysiology study using cultured rat hippocampal neurons showed that patients’ antibodies specifically decreased synaptic NMDAR-mediated currents without affecting AMPA receptor-mediated currents (Hughes et al., 2010). The loss of NMDARs eliminates NMDAR-mediated synaptic function, resulting in dysfunctions in learning, memory and other behavioral aspects. Both *in vivo* and *in vitro* experiments demonstrated that the CSF from patients with anti-NMDAR encephalitis caused a significant decrease in the cell-surface dopamine D_1_ receptor and an increase in D_2_ receptor clusters, and was accompanied by memory impairment and a reduction of surface NMDARs (Carceles-Cordon et al., 2020). The pathogenesis of sleep disorders in anti-NMDAR encephalitis remains to be elucidated. Microinjection of glutamate and NMDA into the rat tuberomammillary nucleus (TMN), which plays a pivotal role in the regulation of sleep-wake patterns, can increase wakefulness and decrease NREM sleep (Yin et al., 2019). Interestingly, some clinical features seen in anti-NMDAR encephalitis are similar to the effect of phencyclidine (PCP) (Mozayani, 2003), an NMDAR antagonist, which can induce hallucinations, seizures and sleep disruption. The neuropsychiatric symptoms of PCP intoxication may be associated with reducing GABAergic transmission *via* NMDAR blockade and activating intracellular endoplasmic reticulum-associated signal transduction, resulting in the enhancement of monoaminergic transmission in the prefrontal cortex (Zhu et al., 2004). Animal models of sleep disorders established using anti-NMDAR antibodies are important for future research. Insomnia that usually presents early in the acute stage is accompanied by agitation, seizures and dysautonomia. This may be because a decrease in NMDARs inactivates the GABAergic neurons, which leads to disinhibition of the excitatory pathways and increase of extracellular glutamate (Dalmau et al., 2011). Hypersomnia during recovery may also present as part of the disease or a related side effect of antiepileptic drugs and antidepressants (Arino et al., 2020). Treatment of anti-NMDAR encephalitis includes first-line immunotherapy (steroids, intravenous immunoglobulin, and plasmapheresis), second-line immunotherapy (rituximab and cyclophosphamide) and tumor removal (Titulaer et al., 2013). Most patients respond to immunotherapy, and second-line immunotherapy is usually effective when the first-line treatments fail. Sleep disturbances such as insomnia and dream-enactment behavior can be improved after immunomodulatory therapies (Blattner et al., 2019).

## Anti-IgLON5 Disease

Anti-IgLON5 disease is a recently reported neurological disorder characterized by unique NREM and rapid eye movement (REM) parasomnia with sleep breathing dysfunction, as well as gait instability and brainstem symptoms (Sabater et al., 2014). Anti-IgLON5 disease does not show sex predominance and usually begins in the sixth decade of life (range: 42–81 years) (Gaig et al., 2018). Human leukocyte antigen (HLA) DRB1^∗^10:01 and HLA-DQB1^∗^05:01 are positive in 87% of patients, and the calculated risk ratio indicated that DRB1^∗^10:01 was 36 times more frequent in patients who developed anti-IgLON5 disease than in the general population (Gaig et al., 2017), and that DRB1^∗^10:01-positive patients developed more frequent sleep symptoms (Gaig et al., 2019a). IgLON5 proteins are highly glycosylated immunoglobulin cell-adhesion molecules that attach to plasma membranes *via* a glycosylphosphatidylinositol anchor (Karagogeos, 2003).

The clinical manifestations of anti-IgLON5 disease are very heterogeneous. Sleep disturbance is a prominent symptom that presents as a complex sleep pattern characterized by abnormal sleep initiation with undifferentiated NREM sleep or poorly structured stage N2, RBD, periods of normal NREM sleep, stridor, and obstructive apnea (Sabater et al., 2014; Gaig et al., 2019b). All 22 patients in a retrospective clinical analysis eventually developed parasomnia, sleep apnea, insomnia or excessive daytime sleepiness (EDS) (Gaig et al., 2017). Undifferentiated NREM sleep is defined by irregularly slow theta EEG activity and the absence of vertex sharp waves, K complexes, sleep spindles, delta slowing, and definite and recurrent REMs, such as those typically seen in REM sleep. Poorly structured stage N2 is characterized by definite K complexes or spindles at 12–14 Hz, which is associated with excessive electromyography (EMG) activity, movements or occasional bursts of REMs of lower amplitude than those typically seen in REM sleep in the same patient. REM sleep is absent or present only in the form of RBD. Obstructive sleep apnea and stridor are also common in these patients (Sabater et al., 2014). Other neurological symptoms include bulbar dysfunction such as dysphagia, dysarthria, sialorrhea, acute respiratory insufficiency, movement disorders, oculomotor abnormalities, cognitive impairment, and autonomic dysfunction (Heidbreder and Philipp, 2018).

The pathophysiology of anti-IgLON5 disease is unclear. The protein function and pathophysiological function of IgLON5 remain unknown. However, despite this lack of clarity, autopsy studies have revealed the absence of inflammatory infiltrates and the presence of neuronal loss, as well as moderate gliosis and extensive neuronal deposits of abnormally hyperphosphorylated three-repeat and four-repeat tau isoforms, preferentially in the hypothalamus and tegmentum of the brainstem, two structures that are crucial for sleep-wake regulation (Sabater et al., 2014; Gelpi et al., 2016). Autoantibodies binding to IgLON5 and tau proteins deposit in the brain indicating the combination of autoimmune and neurodegenerative pathogenesis; however, the primary event is still debatable. A recent case report suggests that tauopathy may be a late, secondary event (Erro et al., 2020). In this study, a 71-year-old male presented with sleep disturbance and bulbar symptoms, and IgLON5 antibodies were detected in the serum and CSF. Histology showed the absence of p-Tau deposits in the brainstem. The pathological changes in the hypothalamus and tegmentum may contribute to sleep problems in anti-IgLON5 disease. A study has reported that IgLON5 clusters were irreversibly decreased in cultured rat hippocampal neurons exposed to the IgLON5 antibodies of patients (Sabater et al., 2016). Recently, IgLON5 IgG was shown to disrupt cytoskeletal organization in cultured rat hippocampal neurons, resulting in dystrophic neurites and axonal swelling (Landa et al., 2020). IgLON5 antibodies may lead to abnormal accumulation of neurofilaments and neurodegeneration by disrupting the crosstalk between the outside of the cell and the cytoskeleton. Most patients were unresponsive to immunotherapy and showed progressive impairment (Sabater et al., 2014). However, Brunetti et al. (2019) reported a case study where immunotherapy with IV immunoglobulins and azathioprine could improve REM sleep and sleep organization with a decrease in the IgLON5-IgG titer, suggesting that IV immunoglobulins may be the first-line therapy for this condition (Logmin et al., 2019). Further studies are required to clarify the effects of immunotherapy. Positive airway pressure treatments may improve respiratory indices in patients with obstructive apnea.

## Anti-Leucine-Rich Glioma Inactivated 1 Encephalitis

Leucine-rich glioma inactivated 1 (LGI1) is a glycoprotein located in the synapse and is expressed mainly in the hippocampus and neocortex (Irani et al., 2010). It is a subunit of presynaptic Kv1-voltage-gated potassium channels. Anti-LGI1 antibodies are commonly found in adult limbic encephalitis (LE). LE presents with acute to subacute onset seizures, memory loss, confusion and other psychiatric symptoms. Approximately 80% of patients with anti-LGI1 antibodies have pathognomonic faciobrachial dystonic seizures (FBDs), which are stereotypical, brief, posturing movements of the hand and ipsilateral hemi-face that are characterized by their strikingly high frequency (Varley et al., 2018). Serum hyponatremia is indicative of the presence of anti-LGI1 antibodies. A study has reported high T2 and fluid-attenuated inversion recovery signals in the bilateral temporal lobe and hippocampus in brain MRI (Wang et al., 2017), which are consistent with the findings that LGI1 is mainly expressed in the temporal cortex and hippocampus. Dysfunction of temporal cortex and hippocampus may be related to temporal epilepsy and cognitive deficits in anti-LGI1 encephalitis patients. Sixty-five percent of patients presented with insomnia, and none of the patients scored high on the Epworth Sleepiness Scale, which is a measure of daytime sleepiness (van Sonderen et al., 2016b). Fifty-seven percent of patients reported dream-enactment behaviors (Blattner et al., 2019). A case report with sequential PSG assessment shows a drastic reduction in total sleep time with complete loss of REM sleep and alterations of slow-wave sleep (Peter-Derex et al., 2012). Delta waves disappeared, whereas few sleep spindles and K-complexes persisted. Recently, a retrospective study based on PSG found that patients with anti-LGI1 encephalitis demonstrated a decrease in total sleep time, sleep efficiency, N3 sleep and REM sleep (Lin et al., 2020). RBD, periodic limb movements in sleep (PLMS) and obstructive sleep apnea were also observed in anti-LGI1 encephalitis. Animal experiments reveal that LGI1 antibody plays a neurotoxic role, which may be mediated through the induction of apoptosis and reduction in calcium currents (Aysit-Altuncu et al., 2018). The LGI1 gene is expressed wildly in the hypothalamus, including the ventromedial nucleus (Herranz-Perez et al., 2010), which contains glycine/GABA neurons and receives direct synaptic input from the glutamatergic neurons in the sublaterodorsal tegmental nucleus. Silencing this circuit was found to induce REM sleep without atonia (Uchida et al., 2020). Antibodies binding hypothalamic neurons may induce hypothalamic dysfunction, likely contributing to RBD and insomnia. Immunomodulatory treatment may improve clinical and PSG outcomes (Peter-Derex et al., 2012; Lin et al., 2020).

## Anti-Contactin-Associated Protein 2 Encephalitis

Anti-contactin-associated protein 2 (CASPR2) antibodies are most common in patients with peripheral nerve hyperexcitability, including those with rare Morvan syndrome (Lim et al., 2015). CASPR2 is a neuronal cell molecule localized at the juxtaparanodes in the myelinated axons (Irani et al., 2010) and is crucial in the clustering of Kv1.1 and Kv1.2 at the juxtaparanode (Gu and Gu, 2011). Morvan syndrome is a rare disorder in which both the peripheral and CNSs are affected. Both anti-CASPR2 and anti-LGI1 antibodies are found in patients with Morvan syndrome, and the anti-CASPR2 antibody titer is higher than that of anti-LGI1 antibodies (Irani et al., 2012; Nikolaus et al., 2018). The clinical manifestations include neuromyotonia, neuropsychiatric features, neuropathic pain and dysautonomia.

Sleep disorders are also common in patients with anti-CASPR2 encephalitis, and 57% of patients showed insomnia in a research study (Joubert et al., 2016; van Sonderen et al., 2016a). Continuous insomnia was more common in patients with anti-CASPR2 encephalitis than with anti-LGI1 encephalitis. Dream-enactment behavior was reported during the acute phase. PSG showed higher limb movement in sleep indexes and status dissociates (Lin et al., 2020). Morvan syndrome has been commonly reported in patients with anti-CASPR2 encephalitis. Insomnia is obvious and prominent. Some patients even report an almost complete absence of sleep (Cornelius et al., 2011). Another review found that the occurrence of severe insomnia was 86% in Morvan syndrome (Abou-Zeid et al., 2012), and PSG results showed reduced or absent sleep spindles and K complexes (typical features of stage 2 NREM sleep). Insomnia in Morvan syndrome was also found to be characterized by REM sleep without atonia and absent or severely reduced slow-wave sleep. An intense reduction of sleep spindles and K complexes and the transient loss of REM sleep atonia indicated agrypnia excitata rather than REM sleep behavior disorder (Vale et al., 2017). Agrypnia excitata is a clinical syndrome characterized by loss of sleep, and autonomic and motor hyperactivation. It has been documented in three different clinical conditions, namely fatal familial insomnia (FFI), Morvan syndrome and delirium tremens (Provini, 2013). The thalamus is the most severely affected brain structure in FFI. Glutamatergic neurons of the paraventricular thalamus (PVT) exhibit high activity during wakefulness, and suppression of PVT neuronal activity causes a reduction in wakefulness, indicating that PVT is a key wakefulness-controlling nucleus (Ren et al., 2018). Functional blockade of the thalamolimbic circuits regulating the sleep-wake cycle has been suggested to contribute to agrypnia excitata (Baldelli and Provini, 2019). Complex behaviors, such as stereotypical rubbing of the hands and crossing the legs, have also been reported (Vale et al., 2017). Anti-CASPR2 antibodies interact with CASPR2, a membrane protein with a large extracellular sequence consisting of multiple domains co-localized with voltage-dependent potassium channels (Poliak et al., 2003). CASPR2 plays a role in maintaining K + channels in the juxtaparanodal region. Kv3 type potassium channel-deficit mice present with sleep loss and increased motor drive, indicating that absence of the Kv3 channel subunits is primarily responsible for the reduction in sleep time (Espinosa et al., 2004). Alleviation of sleep dysfunction after immunotherapy (Cornelius et al., 2011; Peter-Derex et al., 2012) is suggestive of functional changes in the neural circuit instead of irreversible structural impairment in the CNS. Immunotherapy includes treatment with immunoglobulins, corticosteroids and plasmapheresis. Cyclophosphamide can be used to treat anti-CASPR2 encephalitis.

## Anti-Ma2 Paraneoplastic Encephalitis

Ma1 and Ma2 are both onconeuronal target antigens associated with paraneoplastic syndromes. Anti-Ma antibodies recognize Ma1 and Ma2 proteins and are related to paraneoplastic cerebellar degeneration and brainstem encephalitis in various tumors. Anti-Ma2 antibodies react exclusively with Ma2 and are predominantly associated with limbic and brainstem dysfunction in testicular cancer (Voltz et al., 1999). They can be detected in serum and CSF, and serum titers may be correlated to neurologic symptoms (Blumenthal et al., 2006). The clinical manifestations include isolated or combined limbic encephalopathy, and diencephalic or brainstem dysfunction. Sleep disturbances in anti-Ma2 paraneoplastic encephalitis include hypersomnia, RBD and cataplexy. Somnolence is common in anti-Ma2-associated paraneoplastic neurological syndromes (Ortega Suero et al., 2018). Sleep disorders, including narcolepsy and RBD, have also been reported (Adams et al., 2011), and other clinical symptoms include memory deficits, ataxia and seizures. Thirty-two percent of patients showed EDS, and two of them exhibited cataplexy and hypnagogic hallucinations (Dalmau et al., 2004). Patients with EDS have very low or undetectable CSF hypocretin levels (Overeem et al., 2004), which are normal in individuals without EDS. Hypocretin, also known as orexin, plays a vital role in wake regulation (Shan et al., 2015). Hypocretin neurons are mainly located in the lateral hypothalamus (LH). Approximately 90% of hypocretin neurons are lost in narcolepsy, a disease that occurs in the setting of autoimmune encephalitis and characterized by EDS, impaired night-time sleep, sleep paralysis and cataplexy. Neuropathological and immunohistochemistry results showed inflammation and tissue injury exclusively in the hypothalamus in a patient with anti-Ma-associated diencephalitis who presented sleepiness, cataplexy and RBD (Dauvilliers et al., 2013). Cytotoxic CD8 + T lymphocyte-mediated responses against hypocretin neurons may contribute to hypocretin deficiency and result in narcolepsy. Severe hypersomnia, and RBD and narcoleptic features have also been reported in a patient with anti-Ma2 paraneoplastic encephalitis (Compta et al., 2007). Hypothalamus dysfunction might cause sleep disturbance in anti-Ma2 paraneoplastic encephalitis. Oncological therapy, immunotherapy and wakefulness-promoting agents such as modafinil may be effective (Adams et al., 2011).

## Anti-Dipeptidyl-Peptidase-Like Protein 6 Encephalitis

Boronat et al. (2013) first described anti-DPPX encephalitis, a rare autoimmune encephalitis characterized by CNS hyperexcitability (agitation, myoclonus, tremor, and seizures), pleocytosis and frequent diarrhea at symptom onset. Cognitive and mental alterations are common in this condition (Hara et al., 2017). Patients with anti-DPPX encephalitis present with sleep problems, progressive cognitive decline and psychiatric problems, and can be treated using immunotherapy (Zhou et al., 2020). Tobin et al. (2014) described brain or brainstem manifestations of 20 patients with anti-DPPX encephalitis and found disrupted sleep in nine patients: insomnia in six, periodic limb movements in five, sleep apnea in three and hypersomnia in two. Only one patient each had obstructive sleep apnea and ambiguous sleep (REM and NREM sleep occurring simultaneously). PSG results showed ambiguous sleep characterized by REM and NREM sleep occurring simultaneously. DPPX is a regulatory subunit of the Kv4.2 potassium channel complex responsible for transient, inhibitory currents in the central and peripheral nervous system (Varley et al., 2018). Incubation of hippocampal neurons with serum and purified IgG from patients resulted in the decreased expression of DPPX and Kv4.2 in neuronal membranes (Piepgras et al., 2015), which may induce CNS hyperexcitability. *In vivo* studies show that DPPX-KO mice lack the normal hippocampal dendritic A-type K current gradient and that dendrites are hyperexcitable (Sun et al., 2011). Decreased DPPX and Kv4.2 in neuronal membranes may be a pathogenic effect of anti-DPPX antibodies, inducing CNS hyperexcitability such as insomnia. The antibody-induced decrease in DPPX and Kv4.2 protein levels in cultured neurons could be reversed by the removal of the antibodies, indicating the responsiveness of the disorder to immunotherapy (Hara et al., 2017).

## Influenza- and Virus-Related Narcolepsy

Influenza is a viral infection that can induce functional disturbances, such as daytime somnolence, in the CNS. An increased incidence of narcolepsy was associated with the 2009-2010 H1N1 influenza pandemic. Narcolepsy occurred after many days or months. Han et al. (2011) reported a threefold increase in narcolepsy during the 2009 H1N1 winter influenza pandemic. A case-control study of the environmental risk factors of narcolepsy revealed that influenza was a significant risk factor (Picchioni et al., 2007). H1N1 influenza infection in mice lacking B and T cells was found to lead to narcoleptic-like sleep-wake fragmentation and sleep structure alterations, including increased state episodes and stage transitions and decreased REM sleep latency (Tesoriero et al., 2016). Four weeks after infection, an EEG spectral power analysis showed increased slow-wave sleep θ-band, which was similar to that observed in a conditional ablation model of hypocretin deficiency (Tabuchi et al., 2014). The mechanism of influenza-induced narcolepsy is complex. After direct contact with the mucous membranes of the nasal cavity, viruses invade the olfactory cells at the epithelial surface and are anterogradely transported into the olfactory bulb. Next, the viruses can be taken up by afferent terminals and transported retrogradely along the axons of the hypocretin neurons in the LH (Tesoriero et al., 2016). Moreover, infected neurons have been found in the cholinergic basal forebrain (BF), histaminergic TMN, dopaminergic ventral tegmental area (VTA), serotonergic dorsal raphe nucleus (DRN) and noradrenergic locus coeruleus (LC), which are all important wake-related brain areas in the regulation of sleep and wakefulness (Brown et al., 2012; Scammell et al., 2017). The secondary immune reaction after viral infection may contribute to sleep disturbances.

Epidemiological data show that the risk of narcolepsy increased in children and adolescents treated with Pandemrix vaccine (Sarkanen et al., 2018). An increase in narcolepsy diagnoses following the start of AS03-adjuvanted Pandemrix vaccine was observed in Sweden and Finland (Wijnans et al., 2013). The incidence of narcolepsy was 25 times higher after vaccination than before, indicating that Pandemrix vaccination may be a precipitating factor for narcolepsy, especially in combination with HLA-DQB1^∗^0602 (Szakacs et al., 2013). Increased narcolepsy occurred before or after vaccination following the influenza pandemic, indicating an H1N1 virus-derived antigen as a likely trigger (Partinen et al., 2014). CD4T cell cross-reactivity between A/H1N1 pdm09 hemagglutinin antigen and hypocretin might have played a role in the etiology of narcolepsy (Cohet et al., 2019). Molecular mimicry with flu antigens and T cell-mediated hypocretin cell loss was found to contribute to narcolepsy (Luo et al., 2018). Cytotoxic CD8 T cells can destroy hypocretin cells, and the neuronal loss induces clinical signs mimicking human narcolepsy (Bernard-Valnet et al., 2016). The diagnosis can be established based on a history of recent vaccination and typical sleep disturbances; however, the treatment is difficult. Immunosuppression with steroids and immunoglobulins and plasmapheresis were not found to be beneficial.

## Anti-Aquaporin-4 Related Sleep Disorders

Neuromyelitis optica (NMO), an inflammatory and demyelinating disorder of the CNS, is characterized by optic neuritis and transverse myelitis. In the CNS, aquaporin-4 (AQP4) is distributed prominently in the spinal cord, optic nerve and brainstem. Anti-AQP4 IgG antibodies are the most important antibodies related to NMO and are usually found in the serum and CSF of most patients with NMO. Hypersomnia has been reported in a patient having anti-AQP4 antibodies (Suzuki et al., 2012). A permanent hypersomnia status related to a coma-like state has been reported, and the change in sleep architecture may have been caused by damage to the hypocretinergic hypothalamus and a decrease in hypocretin (Carlander et al., 2008). A retrospective analysis shows that patients with NMO had significantly lower serum orexin-A levels than those with narcolepsy as well as healthy controls (Kucukali et al., 2014). Orexin-A levels in the CSF were markedly or moderately reduced (Kanbayashi et al., 2009). A patient with a hypothalamic lesion even presented hypersomnolence as an initial symptom (Deguchi et al., 2012). In another case, EDS was observed in a 41-year-old woman who had an isolated hypothalamic lesion (Sekiguchi et al., 2011). Symptomatic narcolepsy was even listed as one of the core clinical characteristics as the diagnostic criteria for NMO spectrum disorders (Wingerchuk et al., 2015). These findings indicate the functional relationship between AQP4 and hypothalamic damage. AQP4 is expressed throughout the brain, especially in the periaqueductal and periventricular areas, including the hypothalamus. Overall, sleep disturbance in patients with NMO usually features hypersomnia, and the pathological mechanism might be attributed to hypocretinergic hypothalamus involvement. High-dose glucocorticoids and plasma replacement are useful treatment options. Although data from published cases indicate that glucocorticoids may improve hypersomnia and even increase CSF orexin levels (Deguchi et al., 2012; El Otmani et al., 2018), further research on a larger population is needed.

## Future Directions

Autoimmune neurologic diseases, including autoimmune encephalitis, have become newly recognized diseases in the past few years. The clinical signs vary and likely depend on the type of antibodies. We summarized the clinical features, types of sleep disorders, targets, and underlying mechanisms of autoimmune neurological diseases (Table 1). Sleep disorders are common in these patients and might serve as an important feature to help recognize these disorders or confirm the diagnosis. For example, NREM and REM parasomnia with sleep breathing dysfunction might be the main characteristic of IgLON5 disease. Antibodies that damage the sleep-wake regulation areas of the brain may contribute to sleep disturbances in autoimmune neurologic diseases. Improved identification and treatment of sleep disorders may reduce morbidity associated with AE and improve long-term outcomes (Blattner et al., 2019). Further systematic prospective studies are needed to definitively understand the spectrum of sleep disturbances. The development and use of animal models is crucial to more accurately understand the underlying pathophysiological mechanisms and implications of therapeutic approaches.

**TABLE 1:** Clinical features, type of sleep disturbances, targets, and potential mechanisms of autoimmune neurologic diseases.

	**Clinical features**	**Type of sleep disturbance**	**Target**	**Potential mechanism**
NMDAR	Psychiatric and cognitive symptoms, movement disorders, seizures	Insomnia, hypersomnia, sleep-wake reversal, bad dreams/night terrors, less need for sleep	Ionic glutamate receptor	NMDAR reduction, disinhibition of excitatory pathways and increase in extracellular glutamate Dalmau et al., 2011
IgLON5	Sleep disturbances, bulbar dysfunction, movement disorders, oculomotor abnormalities, cognitive impairment, autonomic dysfunction	Undifferentiated NREM sleep, RBD, obstructive sleep apnea, stridor	Hypothalamus, tegmentum	Neuronal loss, moderate gliosis, tau-protein deposits in the hypothalamus and tegmentum of the brainstem Sabater et al., 2014; Gelpi et al., 2016
LGI1	Limbic encephalitis, seizures, FBDs, cognitive impairment	RBD, insomnia, periodic limb movements in sleep, obstructive sleep apnea	Kv1.1	Bind hypothalamic neurons, hypothalamic dysfunction Herranz-Perez et al., 2010
Caspr2	Neuromyotonia, neuropsychiatric features, neuropathic pain and dysautonomia	Insomnia, Morvan syndrome	Kv1.1/Kv1.2	Voltage-dependent potassium channel dysfunction Espinosa et al., 2004
Ma2	Limbic encephalopathy, diencephalic or brainstem dysfunction	Narcolepsy, severe hypersomnia, RBD	Hypothalamus	Hypocretin deficiency Dauvilliers et al., 2013
DPPX	Agitation, myoclonus, tremor, seizures	Insomnia	Kv4.2	Kv4.2 reduction, CNS hyperexcitability Piepgras et al., 2015
Influenza, vaccine	Chills, fever, fatigue, muscle aches, headaches	Narcolepsy	Wake-related brain areas such as LH, BF, TMN, VTA, DRN, LC	T cell-mediated hypocretin cell loss, wake-related neuron loss Bernard-Valnet et al., 2016
AQP4	Optic neuritis, transverse myelitis	Symptomatic narcolepsy, hypersomnia	Periaqueductal and periventricular areas, hypothalamus	Hypothalamic lesion Sekiguchi et al., 2011

## Author Contributions

DY drafted the manuscript. SC and JL revised the manuscript. All authors read and approved the final manuscript.

## Conflict of Interest

The authors declare that the research was conducted in the absence of any commercial or financial relationships that could be construed as a potential conflict of interest.

## Publisher’s Note

All claims expressed in this article are solely those of the authors and do not necessarily represent those of their affiliated organizations, or those of the publisher, the editors and the reviewers. Any product that may be evaluated in this article, or claim that may be made by its manufacturer, is not guaranteed or endorsed by the publisher.
